# Predicting the development of acute kidney injury in liver cirrhosis – an analysis of glomerular filtration rate, proteinuria and kidney injury biomarkers

**DOI:** 10.1111/apt.12299

**Published:** 2013-04-12

**Authors:** A J Slack, M J W McPhail, M Ostermann, M Bruce, R Sherwood, R Musto, T Dew, G Auzinger, W Bernal, J O'Grady, M A Heneghan, K Moore, J A Wendon

**Affiliations:** * Institute of Liver Studies, King's College Hospital Foundation TrustLondon, UK; † Hepatology and Gastroenterology, Division of Diabetes Endocrinology and Metabolism, St Mary's Hospital Campus, Imperial College LondonLondon, UK; ‡ Hepatology, Royal Free HospitalLondon, UK

## Abstract

**Background:**

The timely diagnosis of acute kidney injury (AKI) in liver cirrhosis is challenging.

**Aim:**

To evaluate whether quantification of glomerular filtration rate (GFR), proteinuria and kidney injury biomarkers can accurately predict the development of AKI.

**Methods:**

A prospective cohort analysis of patients with cirrhosis was performed. Measures of baseline kidney function included serum creatinine, iohexol clearance and urine protein:creatinine ratio. Blood and urine samples were collected daily. A retrospective analysis of cystatin C GFR and neutrophil gelatinase-associated lipocalin (NGAL) measured 48 h prior to the diagnosis of AKI was undertaken to evaluate their ability to predict the development of AKI.

**Results:**

Eighteen of the 34 cirrhosis patients studied developed AKI. A GFR <60 mL/min/1.73 m^2^ was identified in 56% with Iohexol clearance compared to 8% using the four-variable modified diet in renal disease formula (*P* < 0.0001). Prediction of AKI, 48 h prior to the development of AKI with cystatin C GFR and serum NGAL concentration were similar; area under the receiver operating curve (AUROC) values 0.74 (0.51–0.97), *P* = 0.04 and 0.72 (0.52–0.92), *P* = 0.02 respectively. The development of AKI was strongly predicted by urine protein:creatinine ratio above the cut-off of >30 (equivalent to 300 mg/day of proteinuria) sensitivity 82% (57–96) and specificity 80% (52–96), AUROC 0.86 (0.73–0.98), *P* ≤ 0.0001. [OR 21 (3–133), *P* ≤ 0.002].

**Conclusions:**

In patients with liver cirrhosis a urine protein:creatinine ratio >30 predicts AKI. Iohexol clearance and cystatin C formulae identify a greater proportion of patients with a GFR <60 mL/min/1.73 m^2^, which also predicts the development of AKI.

## Introduction

**Figure 1:**
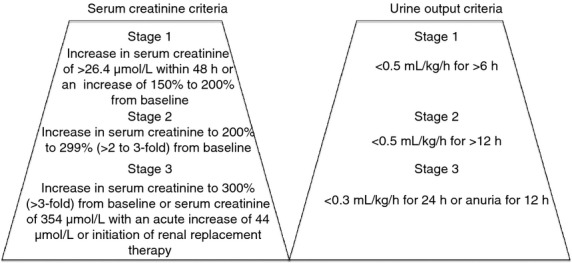
Acute kidney injury network (AKIN) criteria for the definition and classification of AKI.

Acute kidney injury (AKI) is observed in approximately 20% of patients admitted to hospital with decompensated chronic liver disease and ascites.^1^ The definition of AKI has an internationally recognised set of criteria, initially described as the RIFLE criteria,^2^ and subsequently defined by the revised Acute Kidney Injury Network (AKIN) classification and more recently by the Kidney Disease Improving Global Outcomes criteria (Figure 1).^3^, ^4^ Small changes in baseline serum creatinine concentration or urine output form the cornerstone of these classifications with clear clinical consequence of increased morbidity and mortality.

The development and implementation of AKI criteria into clinical practice led to a re-evaluation of how kidney injury should be diagnosed in patients with liver disease. In 2010, a collaboration between the Acute Dialysis Quality Initiative and the International Ascites Club, resulted in proposals for a new term of ‘Hepatorenal dysfunction’, with type II hepatorenal syndrome (HRS) falling within the heading of chronic kidney disease (CKD) whilst type I HRS was recognised as a specific diagnosis within the AKI spectrum.^1^ The quantification of kidney injury in patients with cirrhosis is complicated by differences in analytical methods used to measure serum creatinine and patient factors that change over time influencing measured serum creatinine, such as muscle mass and creatine production, both affected by progressive or advanced liver disease. AKI can therefore develop or be established before changes in creatinine concentration are recognised because serum creatinine remains within the current ‘normal reference range’. For these patients, significant AKI may be seen before the progression to HRS criteria (serum creatinine >133 μmol/L), with the mortality burden associated with AKI.^5^

Earlier identification of at-risk patients and those who have not yet achieved criteria for HRS, may allow interventions to prevent or treat AKI.

Other methods utilised to assess renal function include the estimated glomerular filtration rate (eGFR), applying either the four- or six-variable modified diet in renal disease (MDRD) equation incorporating nonrenal variables to improve accuracy. In patients with cirrhosis, examined pre-transplantation, neither equation performs well in estimating renal function compared with gold standard measures of glomerular filtration rate (GFR) utilising I^125^-iothalamate clearance.^6^ Cirrhosis with established CKD, i.e. GFR <60 mL/min/1.73 m^2^, can go unrecognised in daily clinical practice if estimates of GFR are relied upon.^1^

Assessment of glomerular and/or tubular structure is routinely undertaken in cohorts of renal and diabetic patients with the quantification of proteinuria utilising spot urine protein:creatinine ratio. This has been shown to reflect glomerular (podocyte) structural and/or functional damage, it has important prognostic significance, being strongly linked with progressive decline in kidney function.^7^ It has not been examined in cirrhosis.

A proliferation of studies have been undertaken to evaluate various AKI biomarkers in providing earlier diagnosis of AKI. Most of these studies measured biomarkers after a specific insult (cardiopulmonary bypass^8^ or liver transplantation^9^, ^10^) with relatively few undertaking prospective evaluation in the 48 h prior to onset of AKI.^11^ Two recent studies examined urinary neutrophil gelatinase-associated lipocalin (NGAL) levels in patients with cirrhosis with and without ascites and AKI or HRS and evaluated its role as a predictor of mortality.^12^, ^13^ Urinary NGAL concentrations were significantly higher in those with impaired kidney function, urinary tract infections, CKD and HRS. In those with HRS, urine NGAL concentrations were higher in those with sepsis. Urine NGAL >110 ng/mL predicted mortality (OR >6), adjusting for age and serum creatinine >133 μmol/L.^13^.

Neutrophil gelatinase-associated lipocalin, in both urine and serum, is the most studied biomarker, but others, cystatin C, gamma-glutamyl transpeptidase, interleukin-18 and kidney injury molecule-1 (KIM-1) have also been evaluated and some investigators propose a panel of biomarkers to achieve greater accuracy in earlier diagnosis of AKI.^14^

The aims of this study were threefold to: (i) measure baseline kidney function in a cohort of patients admitted with cirrhosis with a serum creatinine below the upper limit of the normal reference range in the local biochemistry laboratory (<120 μmol/L) and compare with a gold standard measure of GFR; (ii) assess the degree of proteinuria using urine protein:creatinine ratio and (iii) evaluate iohexol GFR and urine protein:creatinine ratio on enrolment, biomarker concentrations measured at both enrolment and 48 h before AKI (changes in serum creatinine or urine output according to AKIN criteria; Figure 1) in predicting the development of AKI.^3^

## Patients and Methods

Between January 2010 and January 2012, we recruited consecutive patients with cirrhosis, admitted either with complications of cirrhosis or for evaluation of suitability for liver transplantation. Entry criteria were the presence of cirrhosis diagnosed on liver biopsy or through clinical, radiographic and biochemistry investigations and a serum creatinine at or below the upper limit of normal, i.e. <120 μmol/L plus one or more of the following; coagulopathy (international normalised ratio >1.5), serum bilirubin > 150 μmol/L or diuretic resistant ascites. Patients with the following criteria, anuria (for 12 h), need for renal replacement therapy (RRT), documented established parenchymal kidney disease or obstructive uropathy based on ultrasound examination were excluded.

Baseline variables were collected, age, gender, morning urine protein:creatinine ratio, expressed as a ratio of urine protein concentration (mg) to urine creatinine concentration (mmol) with a normal ratio <15 (mg/mmol), serum creatinine concentration and eGFR, using the four-variable MDRD equation (=186 × serum creatinine^−1.154^ × age^−0.203^ × (1.212 if black) × (0.742 if female). Baseline serum creatinine concentration was defined as the enrolment serum creatinine as this was available for all patients. Where available we collected pre-enrolment serum creatinine concentrations.

Iohexol clearance determined GFR was calculated by performing a 4-h clearance study. In brief, a bolus of 5 mL of iohexol, concentration 755 mg/mL, was administered at time 0 min with venous sampling at 2, 3 and 4 h. GFR was calculated from the dose divided by the area under the curve.^15^ A high performance liquid chromatography technique, validated in accordance with Food and Drug Administration guidance, was used to determine serum iohexol concentrations.^16^ An iohexol GFR cut-off of <60 mL/min/1.73 m^2^ was used to identify patient with a significantly low GFR. A direct comparison study of this iohexol method was performed against an in-hospital gold standard chromium-51-labelled ethylenediamine tetraacetic acid (^51^Cr-EDTA) in 12 patients with cirrhosis to ensure accuracy and correlation.

Baseline liver-specific variables included aetiology of chronic liver disease, Model for End-Stage Liver Disease (MELD) score^17^ and Child-Pugh (CP) score.1973 Sequential Organ Failure Assessment (SOFA) score was utilised to assess baseline organ function.^19^ To apply the cardiovascular and respiratory SOFA score to ward-based patients, several broad assumptions were necessary. We considered 0.5 and 1 mg of terlipression administered six hourly to be equivalent to a cardiovascular score 3 and 4 respectively. Patients self-ventilating on room air were assumed to have a ventilation score 0. Scores above 0 required arterial blood gas analysis to be available alongside percentage-inspired oxygen.

Daily collection of serum, plasma and urine samples was undertaken to provide aliquots for routine biochemistry analysis; additional aliquots were immediately stored at −80° for subsequent analysis. Physiological variables recorded included heart rate, blood pressure, calculated mean arterial blood pressure and vasopressor use, inclusive of terlipressin use.

Each patient was prospectively followed on a daily basis until the development of AKI according to AKIN criteria (Figure 1).^3^ All data were collected until onset of AKI, discharge from hospital or death over variable time course.

Two AKI biomarkers, serum and urine NGAL and serum cystatin C were evaluated. Serum creatinine and cystatin C concentrations were measured in the main biochemistry laboratory using the modified kinetic Jaffé reaction, which can measure serum creatinine in jaundiced samples^20^ and a latex-enhanced immunoturbidimetric assay^21^ respectively. The cystatin C-based equation of Hoek *et al*.^22^ allowed the calculation of cystatin C GFR from the cystatin C concentrations measured before AKI. NGAL was assayed using a commercially available NGAL Rapid ELISA 037 kit (Bioporto Diagnostics, Gentofte, Denmark).

### Statistical analysis

Continuous data were assessed for normality using the D'Agostino-Pearson test. Measures of central tendency were compared by Student *t-*test (anova) or Mann–Whitney U-test (Kruskal–Wallis) for independent normally or non-normally distributed data. AKI biomarker concentrations collected 48 h prior to the development of AKI were compared with those collected on day 2 of three consecutive daily concentrations in the control group that did not develop AKI. Agreement between enrolment iohexol GFR and eGFR using cystatin C concentrations (Hoek formula) and serum creatinine concentrations (four-variable MDRD formula) measured at 48 h before AKI were compared using Bland–Altman plots.

The relationship between urine protein:creatinine ratio and urine biomarker concentrations was determined. In view of the nonparametric nature of our data, a log transformation was performed before the correlation coefficient using Pearson or Spearman test with scatter diagrams could be performed.

Categorical data were compared using the chi-squared test. The ability of a biomarker to predict an outcome was assessed using the area under the curve generated by receiver operator characteristic (AUROC) analysis. Data were analysed using MedCalc version 10 (MedCalc Software, Mariakerke, Belgium).

### Ethics approval

The Coventry Research and Ethics Committee and the King's College Hospital Research and Development Department approved the study (REC number 09/H1210/72). Patients gave their written informed consent prior to enrolment.

## Results

### Patient baseline characteristics and clinical outcomes

Thirty-four patients with cirrhosis were enrolled and studied. Baseline characteristics are shown in Table 1. The aetiology of cirrhosis was alcohol (37%), immune-mediated (autoimmune hepatitis, primary biliary cirrhosis and primary sclerosing cholangitis) (23%), viral hepatitis (17%), cryptogenic cirrhosis (11%) and miscellaneous causes (12%). All patients had Child-Pugh B or C liver disease, 65% (22 of 34) had grade 2 to 3 ascites.^24^ Of the 34 patients enrolled, 18 (53%) developed AKI based on serum creatinine (*n* = 16) or urine volume criteria (*n* = 2). Ninety-four per cent of the patients (17/18) had stage 1 AKI and one patient (1/18) had stage 3 AKI at the time of sample analysis.

**Table 1:** Baseline characteristics of the 34 chronic liver disease patient

	No-AKI *n* = 16	AKI *n* = 18	*P* value
Baseline variables			
Age (years)	48 ± 12	53 ± 14	0.27
Gender (M:F)	8:12	8:6	0.5
Ascites*	9:7	13:5	0.54
MAP (mm Hg)	82 (10)	79 (14)	0.51
HR (beats/min)	90 (13)	96 (13)	0.2
MELD	18 (13–22)	22 (15–26)	0.28
CP score	11 (9–11)	10 (9–11)	0.86
SOFA score	4 (2)	10 (3)	0.003
Serum creatinine (μmol/L)	68 ± 22	78 ± 20	0.18
eGFR (mL/min/1.73 m^2^)	96 (81–134)	66 (55–96)	0.01
Iohexol GFR (mL/min/1.73 m^2^)†	74 ± 28	52 ± 22	0.02
Cystatin C GFR	73 ± 17	60 ± 22	0.09
Urine protein:creatinine (mg/mmol)	17 (13–29)	55 (36–189)	0.0005
Sodium (mmol/L)	135 ± 6	136 ± 7	0.6
Bilirubin (μmol/L)	94 (51–213)	106 (40–393)	0.65
INR	2.1 (1.5–2.7)	2.0 (1.8–2.5)	0.96
Platelets (×10^9^/L)	112 (78–166)	96 (73–137)	0.27
Albumin (g/L)	31 ± 5	27 ± 7	0.56
CRP (mg/L)	11.5 (6–32)	54 (9–144)	0.07
Outcome			
RRT	0:16	12:6	0.0002
ICU admission	6:12	12:4	<0.05
Alive:Dead	15:1	6:12	0.001
Liver transplantation	9:7	16:2	0.08

AKI, acute kidney injury; CP, Child-Pugh; CRP, C-reactive protein; eGFR, estimated glomerular filtration rate; HR, heart rate; INR, international normalised ratio; MAP, mean arterial pressure; MELD, Model for End-Stage Liver Disease; RRT, renal replacement therapy; SOFA, Sequential Organ Failure Assessment.

Independent *t*-test for parametric data with standard deviation in parenthesis and Mann–Whitney U-test for nonparametric data with interquartile range in parenthesis.

Chi-square for categorical data.

* Grade 2 to 3 ascites defined according to International Ascites Club.^24^

† Twenty-seven iohexol clearance-derived GFR measurements, four patients declined the test.

A similar time course was observed for both specimen collection days between groups (Table 1). The mean lead-time from enrolment to the development of AKI was 4.5 ± 2 days. Baseline serum creatinine concentration, measured on the day of enrolment, correlated well with serum creatinine concentration measured pre-enrolment, *r* = 0.72, *P* = 0.002. There was a median (IQR, interquartile range) of 14 days (6–48) between pre-enrolment and enrolment data points. Median pre-enrolment serum creatinine concentration was 86 μmol/L (74–102) vs. baseline (enrolment) creatinine 74 μmol/L (56–86), *P* = 0.3 (Wilcoxon-paired test).

Eleven of the 18 patients with AKI fulfilled HRS criteria^1^ based on changes in serum creatinine concentration, occurring at a median of 2 (0–2) days after the development of AKI. Of the eleven, six patients developed creatinine-based criteria for HRS, but had proteinuria >500 mg/day assessed by spot urine protein:creatinine ratio >50. AKI insults were multi-factorial involving prerenal insults (9/18), large volume paracentesis (1/18), systemic inflammatory response syndrome with or without detectable bacteraemia (but treated with empirical antibiotic therapy) (8/18). No episodes of postrenal injury were identified.

Sixteen of the 18 patients who developed AKI were admitted to the intensive care unit. Enrolment SOFA scores were significantly higher in those patients who subsequently developed AKI compared with those who did not (Table 1). Although no differences were seen comparing both MELD and CP scores.

Acute kidney injury resolved with medical intervention in four patients suggestive of reversible prerenal aetiology. Twelve patients developed AKI, due to either acute tubular necrosis (7/12) or HRS type 1 (5/12) that required the initiation of RRT. Of these 12 patients that received RRT two recovered kidney function, three underwent liver transplantation with recovery of renal function and seven died of multi-organ dysfunction. The hospital mortality of patients with AKI was 67% compared to 6% among patients without AKI. The leading cause of death was multiple-organ failure.

Baseline serum creatinine and eGFR performed poorly in predicting the development of AKI, with AUROC values of 0.66 (0.46–0.85) with a cut-off of >63 μmol/L (*P* = 0.12), and 0.63 (0.44–0.82) with a cut-off of <92 mL/min/1.73 m^2^ (*P* = 0.19), respectively. Serum creatinine concentration and eGFR displayed improved performance at 48 h prior to the development of AKI with AUROC values of 0.65 (0.46–0.85) with a cut-off of 67 μmol/L and 0.67 (0.48–0.86) with a cut-off of 77 mL/min/1.73 m^2^, *P* = 0.12 and 0.08 respectively.

Baseline iohexol GFR assessment was undertaken in 27 patients entered into the study, seven patients declined. This method showed good accuracy compared with the in-hospital gold standard ^51^Cr-EDTA with a mean difference −1.3 (−18 to +16) mL/min/1.73 m^2^ using Bland–Altman plot analysis. Of these 27 patients undergoing iohexol GFR assessment, 56% had an iohexol GFR <60 mL/min/1.73 m^2^ despite all having a serum creatinine <120 μmol/L.

Estimated GFR was also calculated with both cystatin C GFR (Hoek) formula and four-variable MDRD formula, incorporating cystatin C and serum creatinine concentrations measured 48 h prior to the development of AKI. A GFR <60 mL/min/1.73 m^2^ was identified in (11/29) 38% of patients when using cystatin C-based formula compared to (4/34) 12% with the MDRD formula measured at 48 h prior to the development of AKI.

Bland–Altman analysis of both cystatin C and MDRD formula, used to estimate GFR at 48 h prior to the development of AKI, indicated that the cystatin C-based formula was most accurate with a mean difference (bias) of −2 (−40 to +35) mL/min/1.73 m^2^ compared to iohexol GFR. The four-variable MDRD equation was neither accurate nor precise, displaying a mean difference (bias) of −34 (−92 to +24) mL/min/1.73 m^2^.

Baseline (enrolment) iohexol GFR and cystatin C GFR (measured 48 h prior to AKI according to AKIN criteria) displayed similar ability to predict AKI as judged by AUROC values of 0.73 (0.54–0.93), (cut-off <55 mL/min/1.73 m^2^) *P* = 0.02 and 0.71 (0.5–0.92), (cut-off <58 mL/min/1.73 m^2^), *P* = 0.05 respectively.

Ninety-one per cent of patients (29/32) had baseline proteinuria as defined by an enrolment urine protein:creatinine ratio greater than normal (>15). It was significantly higher in those who developed AKI [AKI median urine protein:creatinine ratio 55 (36–189) vs. No-AKI 17 (13–29), *P* = 0.0005].

Six of thirty-two patients (19%) with alcohol-related or immune-mediated liver disease had a urine protein:creatinine ratio >100, the equivalent of greater than 1 g/day. Two of the six displayed nephrotic range proteinuria (urine protein:creatinine ratio >300) and four had a urine protein:creatinine ratio of between 100 and 200. Statistical significance between the AKI and No-AKI groups was maintained after the omission of patients with both proteinuria, equivalent to greater than 1 g/day and nephrotic range proteinuria *P* ≤ 0.0015 and 0.016.

Proteinuria of greater than 500 mg/day, the equivalent of a protein:creatinine ratio of >50, represents the cut-off for exclusion of HRS, based on current International Ascites Club criteria. The omission of patients with proteinuria above this cut-off resulted in a non-significant difference in median protein:creatinine ratio (IQR) 16.5 (13**–**26) vs. 35 (16**–**40) between the two groups AKI or no-AKI (*n* = 21, *P* = 0.1).

**Figure 2:**
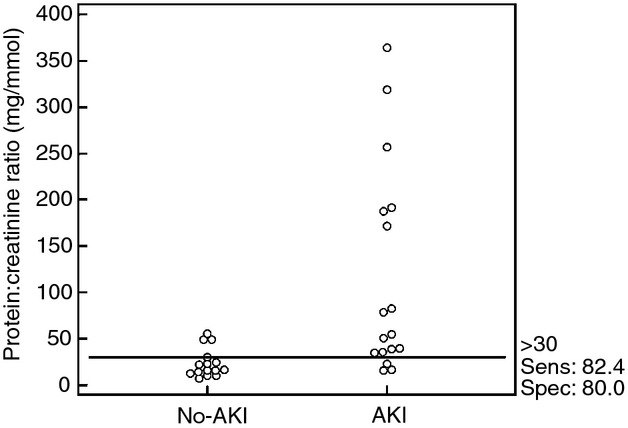
Interactive dot-line diagram: comparison of all protein:creatinine ratio measurements in liver cirrhosis patients with and without acute kidney injury (AKI).

Baseline (enrolment) urine protein:creatinine ratio was strongly predictive of the development of AKI, above a cut-off of 30 (equivalent to 300 mg/day of proteinuria) sensitivity 82% and specificity 80% [OR 21 (3–133), *P* ≤ 0.002], AUROC 0.86 (0.73–0.98), *P* ≤ 0.0001. Seventeen patients (53%) had protein:creatinine ratio >30, of whom 53%, developed AKI compared to only 12% (four patients) who did not develop AKI (Figure 2). The omission of all patients with a protein:creatinine ratio of either >100 or 50 did not affect the cut-off of 30 or significance for predicting AKI, *P* = 0.002 and 0.05.

**Figure 3:**
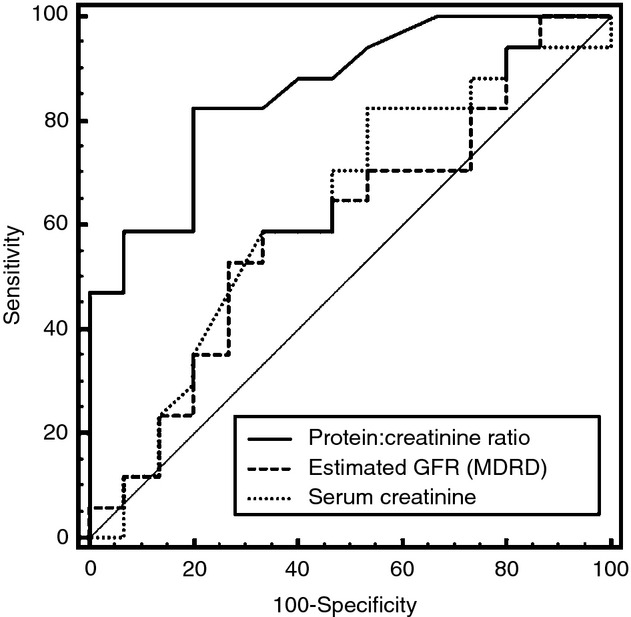
Comparative area under the receiver operating curve (AUROC) of baseline protein:creatinine ratio, serum creatinine and estimated glomerular filtration rate (eGFR) to predict development of acute kidney injury (AKI). An AUROC comparison graph for 32 patients with baseline urine protein:creatinine ratio, serum creatinine concentration and eGFR. Urine protein:creatinine ratio displays good discriminative performance for future AKI insults. When compared directly with baseline serum creatinine and eGFR, there is a statistically significant difference compared to eGFR, *P* = 0.035 and trend towards significance compared to baseline serum creatinine, *P* = 0.07.

A comparative AUROC analysis of baseline measures, including urine protein:creatinine ratio, iohexol GFR, serum creatinine concentration and eGFR at predicting AKI demonstrated urine protein:creatinine ratio to be superior to baseline serum creatinine concentration or eGFR (*P* = 0.07 and 0.035, respectively, Figure 3). Iohexol GFR did not outperform urine protein:creatinine ratio (*P* = 0.1).

### Median sequential biomarker data

Median biomarker concentrations were compared between patients with and without AKI (Table 2). This analysis of cystatin C and NGAL values was performed on samples collected in the days taken up to, but not including the day of AKI.

**Table 2:** A comparison of serum and urine AKI biomarkers, isolated 48 h and median concentrations, before the development of AKI

Biomarker	No-AKI	AKI	*P* value
Baseline serum creatinine (μmol/L)	66 (55–78) *n* = 16	78 (65–88) *n* = 18	0.12
(48 h) Serum creatinine (μmol/L)	66 (50–86) *n* = 16	79 (71–96) *n* = 18	0.13
Baseline eGFR (mL/min/1.73m^2^)	105 (83–115) *n* = 16	89 (66–109) *n* = 18	0.2
(48 h) eGFR	100 (83–133)	77 (67–107)	0.09
Median cystatin C (mg/L)	1.08 (0.28) *n* = 15	1.38 (0.44) *n* = 15	0.04
(48 h) Cystatin C (mg/L)	1.04 (0.85–1.18) *n* = 15	1.28 (1.0–1.8) *n* = 15	0.09
Median serum NGAL (ng/mL)	154 (116–228) *n* = 15	235 (171–510) *n* = 18	0.09
(48 h) Serum NGAL (ng/mL)	143 (94–236)	271 (166–551)	0.05
Median CRP (mg/L)	11 (6–32) *n* = 17	54 (9–144) *n* = 16	0.07
Median urine NGAL (ng/mL)	60 (39–100) *n* = 16	87 (44–144) *n* = 15	0.17
(48 h) Urine NGAL (ng/mL)	65 (32–114)	79 (57–171)	0.17
Median normalised urine NGAL (ng/mg urine creatinine)	78 (48–142) *n* = 10	154 (109–257) *n* = 7	0.028

AKI, acute kidney injury; CRP, C-reactive protein; eGFR, glomerular filtration rate; NGAL, neutrophil gelatinase-associated lipocalin.

Medians expressed with interquartile range (25:75) or mean expressed with standard deviation.

(48 h) – measured 48 h prior to the development of AKI.

Median cystatin C and normalised urine NGAL concentrations prior to AKI insult were significantly different between groups (*P* = 0.04 and 0.028 respectively). Serum NGAL and C-reactive protein (CRP) were higher in the AKI group, but this did not reach statistical significance (*P* = 0.09 and 0.07 respectively). A significant correlation between CRP measured before the development of AKI and serum NGAL was identified, *P* = 0.002. No correlation between SOFA scores and serum NGAL was evident, *P* = 0.4.

Predicting AKI with serum NGAL concentrations measured at 48 h prior to AKI displayed fair discriminative performance with AUROC values of 0.72 [0.52–0.92, *P* = 0.02 (cut-off 241 ng/mL)]. This was superior to either serum creatinine or eGFR.

**Figure 4:**
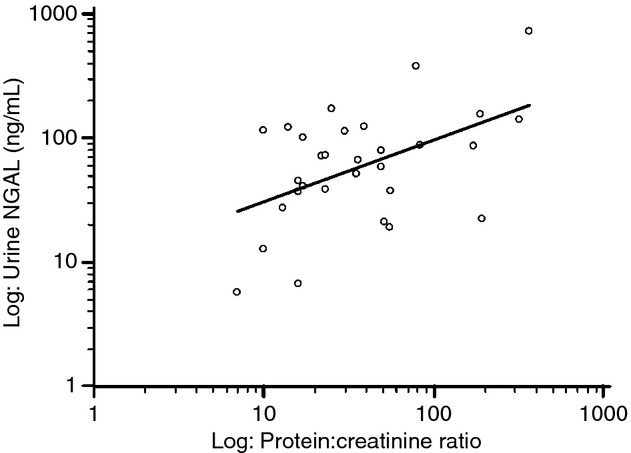
Correlation between the protein:creatinine ratio and urine neutrophil gelatinase-associated lipocalin concentrations.

A positive correlation between urine NGAL and urine protein:creatinine ratio was identified. In view of the nonparametric nature of our data, a log transformation was performed for urine NGAL and urine protein:creatinine ratio [*r* = 0.496 (95% CI 0.164–0.727), *P* = 0.005, Figure 4].

## Discussion

This study prospectively examined a cohort of patients with cirrhosis who at presentation were thought to have normal kidney function (as defined by a serum creatinine below the upper limit of the normal reference range <120 μmol/L) yet considered at high risk of developing AKI.

This study demonstrates that proteinuria is common in patients with cirrhosis (91%) and that the quantification of proteinuria is important. A urine protein:creatinine ratio >30 is both a sensitive and specific marker for predicting hospital AKI and mortality.

An GFR <55 mL/min/1.73 m^2^, measured using a gold standard validated method, iohexol clearance, identifies patients at-risk of AKI. Cystatin C-based formulae for estimating GFR were more accurate and precise compared with the four-variable creatinine-based MDRD formula and identified a greater proportion of patients with a low GFR <60 mL/min/1.73 m^2^. Cystatin C formulae may offer a simpler alternative to gold standard clearance methods for determining GFR in cirrhosis and appear to predict development of AKI when determined at 48 h prior to the insult. Serum cystatin C concentration measured daily may additionally identify patients at-risk of AKI.

The evaluation of proteinuria using the urine protein:creatinine ratio is simple, easily applicable and appears to be a potentially useful test in assessing risk of AKI in patients with cirrhosis. Urine protein:creatinine ratio out-performed other biomarkers and was highly predictive when >30 for the development of AKI. This emphasises the importance of identifying cirrhotic patients with a small degree of proteinuria.

In health, protein is prevented from entering tubular fluid by a complex glomerular barrier, which is usually highly effective.^27^ Glomerular disease results in defects in this barrier leading to substantial increases in urinary protein, which has been shown to contribute to tubular cell apoptosis. This has been linked to the up-regulation of kidney tubular biomarkers, like NGAL and KIM-1.^28^ In liver disease declining kidney function has been associated with increased biomarker concentrations.^29^ In type 1 diabetics, a correlation between low microalbuminuria and lower KIM-1 concentrations has been reported.^30^

The observed high incidence of mild proteinuria in patients with cirrhosis developing AKI requires further investigation. It may represent unrecognised glomerular disease, which is not uncommon in patients with cirrhosis. Hepatic IgA nephropathy is recognised with alcohol-related liver disease and hepatitis C is associated with glomerulonephritis.^31^ Such glomerular diseases are often clinically silent and may explain the observation that the no-AKI group had a median protein:creatinine ratio greater than 15. Equally it may represent a marker of early decline in renal function as seen in diabetic patients. The finding of low level proteinuria even in those who did not develop AKI on that admission may suggest a need for regular screening and quantification for proteinuria in both out-patient and in-patient populations.

Serum NGAL performed in line with a previously published meta-analysis where cut-off and AUROC values, 25**–**550 ng/mL and AUROC 0.71**–**0.93 were reported.^32^ We also demonstrated a correlation between serum NGAL and CRP, but not SOFA scores in the AKI group. The exact reasons are not clear. However, it is possible that the association with CRP is due to underlying inflammation and/or sepsis which may have occurred before the onset of organ dysfunction.^33^ It may also suggest that inflammation has a greater influence on both serum and urine NGAL biomarker levels than organ dysfunction. High serum NGAL concentration will directly affect urine NGAL concentrations as it is freely filtered at the glomerulus. A ratio of urine to serum NGAL concentrations has been suggested as a method to correct for this effect.

There are important clinical implications attached to the methods chosen to accurately evaluate baseline kidney function. This will affect both the screening and management of patients with cirrhosis, in the out-patient setting, at admission with acute decompensation or at assessment for transplantation. It may also influence decisions regarding the institution of specific treatment for impaired renal function, immunosuppression regimens and prospects of combined liver and kidney transplantation. The observation that GFR, renal blood flow and urinary sodium clearance increases with terlipressin given to patients with a normal creatinine and ascites that was not diuretic resistant highlights its role as a potential therapeutic agent prior to the onset of recognised HRS.^34^ Earlier identification of patients at-risk, i.e. with low GFRs and mild degrees of proteinuria may allow timely decisions regarding withdrawal of diuretic therapy, volume challenge with an appropriate fluid and terlipressin administration to prevent AKI or to reduce its magnitude.

All of our patients received gelatine-based fluid and albumin for volume resuscitation; none received starch-based solutions. The choice of fluid in resuscitation has recently been highlighted as an important factor given the adverse effects on kidney function and increased requirement for RRT associated with hydroxyethyl starch-based fluid.^35^

The major weakness of this study is the low number of patients followed sequentially, and incomplete data points. The use of AKIN criteria for the diagnosis of AKI has only recently been proposed for the use in patients with cirrhosis.^1^ Single daily collections of blood and urine may not be frequent enough given biomarkers like NGAL are up regulated within hours of AKI. Finally, single measurement of urine protein:creatinine ratio at enrolment did not enable us to establish either the persistence of proteinuria or its pre-existing level. Urine protein:creatinine ratio should be considered to be included as part of a routine screening tool, in patients with cirrhosis, to risk stratify those patients who are at increased risk of AKI.

In conclusion, urine protein:creatinine ratio appears to be a sensitive, specific and an easily applicable marker of risk for the development of AKI in liver cirrhosis. A protein:creatinine ratio >30 is a significant risk factor for AKI. Similarly, an iohexol GFR or cystatin C-based GFR <60 mL/min/1.73 m^2^ is associated with an increased risk of AKI. Our results show that eGFR formulae using cystatin C are more accurate than those that incorporate creatinine concentration and may offer a simpler alternative to gold standard GFR clearance studies.

## References

[1] Wong F, Nadim MK, Kellum JA (2011). Working party proposal for a revised classification system of renal dysfunction in patients with cirrhosis. Gut.

[2] Hoste EA, Kellum JA (2006). RIFLE criteria provide robust assessment of kidney dysfunction and correlate with hospital mortality. Crit Care Med.

[3] Mehta RL, Kellum JA, Shah SV (2007). Acute kidney injury network: report of an initiative to improve outcomes in acute kidney injury. Crit Care.

[4] Khwaja A (2012). KDIGO clinical practice guidelines for acute kidney injury. Nephron Clin Pract.

[5] Mucino-Bermejo J, Carrillo-Esper R, Uribe M, Mendez-Sanchez N (2012). Acute kidney injury in critically ill cirrhotic patients: a review. Ann Hepatol.

[6] Gonwa TA, Jennings L, Mai ML, Stark PC, Levey AS, Klintmalm GB (2004). Estimation of glomerular filtration rates before and after orthotopic liver transplantation: evaluation of current equations. Liver Transpl.

[7] Wu MT, Lam KK, Lee WC (2012). Albuminuria, proteinuria, and urinary albumin to protein ratio in chronic kidney disease. J Clin Lab Anal.

[8] Wagener G, Gubitosa G, Wang S, Borregaard N, Kim M, Lee HT (2008). Urinary neutrophil gelatinase-associated lipocalin and acute kidney injury after cardiac surgery. Am J Kidney Dis.

[9] Portal AJ, McPhail MJ, Bruce M (2010). Neutrophil gelatinase-associated lipocalin predicts acute kidney injury in patients undergoing liver transplantation. Liver Transpl.

[10] Wagener G, Minhaz M, Mattis FA, Kim M, Emond JC, Lee HT (2011). Urinary neutrophil gelatinase-associated lipocalin as a marker of acute kidney injury after orthotopic liver transplantation. Nephrol Dial Transplant.

[11] Nejat M, Pickering JW, Devarajan P (2012). Some biomarkers of acute kidney injury are increased in pre-renal acute injury. Kidney Int.

[12] Fagundes C, Pepin MN, Guevara M (2012). Urinary neutrophil gelatinase-associated lipocalin as biomarker in the differential diagnosis of impairment of kidney function in cirrhosis. J Hepatol.

[13] Verna EC, Brown RS, Farrand E (2012). Urinary neutrophil gelatinase-associated lipocalin predicts mortality and identifies acute kidney injury in cirrhosis. Dig Dis Sci.

[14] Lisowska-Myjak B (2010). Serum and urinary biomarkers of acute kidney injury. Blood Purif.

[15] Nilsson-Ehle P, Grubb A (1994). New markers for the determination of GFR: iohexol clearance and cystatin C serum concentration. Kidney Int Suppl.

[16] Shah VP, Midha KK, Findlay JW (2000). Bioanalytical method validation – a revisit with a decade of progress. Pharm Res.

[17] Malinchoc M, Kamath PS, Gordon FD, Peine CJ, Rank J, ter BorgPC (2000). A model to predict poor survival in patients undergoing transjugular intrahepatic portosystemic shunts. Hepatology.

[18] Pugh RN, Murray-Lyon IM, Dawson JL, Pietroni MC, Williams R (1973). Transection of the oesophagus for bleeding oesophageal varices. Br J Surg.

[19] Vincent JL, Moreno R, Takala J (1996). The SOFA (Sepsis-related Organ Failure Assessment) score to describe organ dysfunction/failure. On behalf of the Working Group on Sepsis-Related Problems of the European Society of Intensive Care Medicine. Intensive Care Med.

[20] Hervey GR (1953). Determination of creatinine by the Jaffe reaction. Nature.

[21] Lewis AV, James TJ, McGuire JB, Taylor RP (2001). Improved immunoturbidimetric assay for cystatin C. Ann Clin Biochem.

[22] Hoek FJ, Kemperman FA, Krediet RT (2003). A comparison between cystatin C, plasma creatinine and the Cockcroft and Gault formula for the estimation of glomerular filtration rate. Nephrol Dial Transplant.

[23] Moore KP, Wong F, Gines P (2003). The management of ascites in cirrhosis: report on the consensus conference of the International Ascites Club. Hepatology.

[24] Toblli JE, Bevione P, Di GennaroF, Madalena L, Cao G, Angerosa M (2012). Understanding the mechanisms of proteinuria: therapeutic implications. Int J Nephrol.

[25] Waanders F, Vaidya VS, van GoorH (2009). Effect of renin-angiotensin-aldosterone system inhibition, dietary sodium restriction, and/or diuretics on urinary kidney injury molecule 1 excretion in nondiabetic proteinuric kidney disease: a post hoc analysis of a randomized controlled trial. Am J Kidney Dis.

[26] Gerbes AL, Benesic A, Vogeser M, Krag A, Bendtsen F, Moller S (2011). Serum neutrophil gelatinase-associated lipocalin – a sensitive novel marker of renal impairment in liver cirrhosis?. Digestion.

[27] Vaidya VS, Niewczas MA, Ficociello LH (2011). Regression of microalbuminuria in type 1 diabetes is associated with lower levels of urinary tubular injury biomarkers, kidney injury molecule-1, and N-acetyl-beta-D-glucosaminidase. Kidney Int.

[28] Pouria S, Feehally J (1999). Glomerular IgA deposition in liver disease. Nephrol Dial Transplant.

[29] Haase M, Bellomo R, Devarajan P, Schlattmann P, Haase-Fielitz A (2009). Accuracy of neutrophil gelatinase-associated lipocalin (NGAL) in diagnosis and prognosis in acute kidney injury: a systematic review and meta-analysis. Am J Kidney Dis.

[30] Cowland JBMT, Borregaard N (2006). IL-1 beta-specific up-regulation of neutrophil gelatinase-associated lipocalin is controlled by IkappaB-Zetha. J Immunol.

[31] Krag A, Moller S, Henriksen JH, Holstein-Rathlou NH, Larsen FS, Bendtsen F (2007). Terlipressin improves renal function in patients with cirrhosis and ascites without hepatorenal syndrome. Hepatology.

[32] Myburgh JA, Finfer S, Bellomo R (2012). Hydroxyethyl starch or saline for fluid resuscitation in intensive care. N Engl J Med.

